# Latest Developments in Adapting Deep Learning for Assessing TAVR Procedures and Outcomes

**DOI:** 10.3390/jcm12144774

**Published:** 2023-07-19

**Authors:** Anas M. Tahir, Onur Mutlu, Faycal Bensaali, Rabab Ward, Abdel Naser Ghareeb, Sherif M. H. A. Helmy, Khaled T. Othman, Mohammed A. Al-Hashemi, Salem Abujalala, Muhammad E. H. Chowdhury, A.Rahman D. M. H. Alnabti, Huseyin C. Yalcin

**Affiliations:** 1 Electrical and Computer Engineering Department, The University of British Columbia, Vancouver, BC V6T 1Z4, Canada; anas5@student.ubc.ca (A.M.T.); rababw@ece.ubc.ca (R.W.); 2 Biomedical Research Center, Qatar University, Doha 2713, Qatar; onur.mutlu@qu.edu.qa; 3 Department of Electrical Engineering, Qatar University, Doha 2713, Qatar; f.bensaali@qu.edu.qa (F.B.); mchowdhury@qu.edu.qa (M.E.H.C.); 4 Heart Hospital, Hamad Medical Corporation, Doha 3050, Qatar; aallam4@hamad.qa (A.N.G.); kothman2@hamad.qa (K.T.O.); sma1200@gmail.com (S.A.); 5 Faculty of Medicine, Al Azhar University, Cairo 11884, Egypt; 6 Noninvasive Cardiology Section, Cardiology Department, Heart Hospital, Hamad Medical Corporation, Doha 3050, Qatar; shelmy@hamad.qa (S.M.H.A.H.); malhashemi@hamad.qa (M.A.A.-H.); 7 Department of Biomedical Science, College of Health Sciences, QU Health, Qatar University, Doha 2713, Qatar

**Keywords:** cardiovascular hemodynamics, computational modeling, deep learning, graph convolutional network, transcatheter aortic valve replacement, transcatheter aortic valve implantation

## Abstract

Aortic valve defects are among the most prevalent clinical conditions. A severely damaged or non-functioning aortic valve is commonly replaced with a bioprosthetic heart valve (BHV) via the transcatheter aortic valve replacement (TAVR) procedure. Accurate pre-operative planning is crucial for a successful TAVR outcome. Assessment of computational fluid dynamics (CFD), finite element analysis (FEA), and fluid–solid interaction (FSI) analysis offer a solution that has been increasingly utilized to evaluate BHV mechanics and dynamics. However, the high computational costs and the complex operation of computational modeling hinder its application. Recent advancements in the deep learning (DL) domain can offer a real-time surrogate that can render hemodynamic parameters in a few seconds, thus guiding clinicians to select the optimal treatment option. Herein, we provide a comprehensive review of classical computational modeling approaches, medical imaging, and DL approaches for planning and outcome assessment of TAVR. Particularly, we focus on DL approaches in previous studies, highlighting the utilized datasets, deployed DL models, and achieved results. We emphasize the critical challenges and recommend several future directions for innovative researchers to tackle. Finally, an end-to-end smart DL framework is outlined for real-time assessment and recommendation of the best BHV design for TAVR. Ultimately, deploying such a framework in future studies will support clinicians in minimizing risks during TAVR therapy planning and will help in improving patient care.

## 1. Introduction

In the last two decades, cardiovascular disease (CVD) has become the single and largest cause of non-communicable disease deaths worldwide, which contributes to over 50% of worldwide deaths. The World Health Organization (WHO) estimates that 17.6 million people died of CVDs worldwide in 2012, proportionally accounting for an estimated 31.3% of global mortality [1]. Among CVDs, heart valve defects, particularly aortic valve (AV) defects, are the most prevalent. AV regulates oxygenated blood flow exiting the left ventricle through the aorta to the rest of the body. AV operates under complex and strong hemodynamic forces. Defects in that valve can form congenitally, such as a bicuspid valve, or they might develop later in life, such as valve calcification. The incidence of AV defects increases with age and reaches beyond 3% above the age of 65 [2].

A severely damaged or non-functioning aortic valve is replaced with a mechanical heart valve (MHV) or a bioprosthetic heart valve (BHV). MHVs have enhanced durability but are implanted via invasive surgery and require anticoagulant therapy upon implantation. BHVs can be implanted via open-heart surgery or via a less invasive transcatheter aortic valve replacement (TAVR), which is also known as transcatheter aortic valve implantation (TAVI); the former term is used for the remaining parts of this paper. In current practice, MHVs are recommended for younger patients, and BHVs are recommended for the elderly. With advancements in valve production technologies, BHVs are becoming more popular and applicable to younger and lower-risk patients. With its minimally invasive character, it is expected soon that the majority of valve replacements will be via TAVR.

For a successful TAVR, it is critically important to predict the performance of BHV upon implantation. As there is no direct view or access to the affected anatomy, accurate preoperative planning is crucial for a successful outcome. The most important decisions during planning are selecting the proper implant type/size as well as positioning the valve in the aortic root. Due to the wide variety in valve sizes and types and non-circular annulus shapes, there is often no obvious choice for a given patient. Most clinicians base their final decision on their previous experience. However, it is desirable to mechanically assess selected BHV before implantation to ensure proper valve function with minimal complications after implantation. 

Mechanical assessment refers to being able to realistically simulate the opening and closing of a valve to examine valve function. Such an assessment will prevent improper valve selection that could lead to possible complications, such as paravalvular leaks or conduction problems, which are major issues for TAVR. Assessment of mechanical stresses using computational modeling, such as computational fluid dynamics (CFD) and finite element analysis (FEA), offers a solution that has been increasingly utilized to evaluate native and bioprosthetic valve mechanics. However, such patient-specific models usually require complex procedures to set up and long computing times to obtain final simulation results, preventing prompt feedback to clinicians in time-sensitive clinical applications. A potential practical and effective solution for implementing engineering analysis to the mechanical assessment of BHVs is to incorporate deep learning (DL)-based systems to expedite and simplify the computational biomechanical analyses relevant to TAVR. 

Recently, DL algorithms have been investigated as a fast and computational light alternative to traditional computational methods to compute patient-specific hemodynamics, whereas only very few studies have considered TAVR as an application. Hence, there exists a good room for improvement in addressing the current gaps in the literature. Therefore, in this study, we conduct a comprehensive review of current DL approaches to predict cardiovascular hemodynamics, providing a detailed comparison, highlighting current limitations, and recommending some future directions. First, we introduce the classical medical approaches for TAVR, and we focus on medical imaging modalities. Then, we provide a brief overview of the end-to-end conventional computational modeling pipeline for assessing TAVR outcomes, including geometry segmentation from cardiac computed tomography (CT) or magnetic resonance imaging (MRI), hemodynamic prediction, and derived parameters of clinical relevance. Next, we conduct a comprehensive review of current DL alternative for each stage of the conventional approach, including segmentation, hemodynamic prediction, and pre- and post-operative clinical assessments. Since hemodynamic prediction is the key component in a DL-based system for TAVR, we provide a detailed comparison of related studies, highlighting the utilized datasets, the achieved results, and the deployed models. 

**Figure 1:**
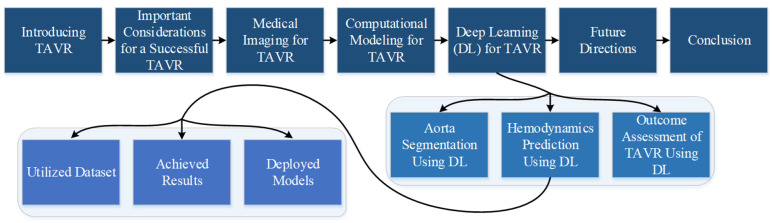
The outline of this review paper.

The rest of the paper is organized as follows. Section 2 briefly reviews the classical approaches for planning and assessing TAVR, highlighting important considerations for successful procedures. Section 3 outlines and compares different medical imaging techniques used for TAVR pre- and post-operative assessments. Section 4 overviews the usage of computational modeling for TAVR procedures, while Section 5 provides a comprehensive review of recent DL approaches for TAVR procedures, starting from aortic segmentation through hemodynamic prediction to treatment planning and TAVR outcome assessment. The current limitations and future directions are discussed in Section 6, and a unified DL framework is outlined for real-time assessment and recommendation of the best BHV design for TAVR. Finally, the conclusions are drawn in Section 7. Figure 1 represents the sections of this review.

## 2. Important Considerations for a Successful TAVR

While conventional MHVs or BHVs are surgically implanted via invasive open-heart surgery, TAVR has been introduced about two decades ago as an alternative for minimally invasive implantation of new-generation BHVs [3]. For TAVR, a stented valve is inserted into the aortic root using a catheter through the femoral, subclavian, or carotid artery. The catheter is guided into the heart through moving X-ray images (fluoroscopy) and echocardiogram. Transcatheter BHVs are implanted through the native leaflets. These valves are implanted while diseased native valves are still in place. The Placement of Aortic Transcatheter Valve (PARTNER) trials demonstrated TAVR superiority in short- and medium-term mortality, which resulted in the establishment of this new revolutionary treatment in the last decade with growing safety and efficacy [4]. Given its promises, TAVR has recently been approved for the intermediate-risk patient population, who will benefit from shorter hospital stays [5], and have even shown superior safety for low-risk patient groups compared to surgical valve replacement [6,7]. In the near future, transcatheter valves are expected to have comparable durability with surgical valves, resulting in the complete replacement of surgical valve replacement therapy with TAVR, as foreseen from a 2006 poll in Europe [3].

Unlike surgical replacement valves in which the valve is sutured to the root and the native valve is removed, in TAVR, a stented BHV is anchored to the aortic root while the native valve is still in place. For this practice, the selection of a proper valve, as well as its implantation position, is of utter importance for success. Major complications following BHV implantations with TAVR include migration of the valve; paravalvular regurgitation between native and implanted valves; excessive pressure from the stent on the root, which may cause conduction anomalies or annular rupture; and coronary obstruction [8]. A small BHV might migrate and result in paravalvular regurgitation, while a large BHV can cause conduction problems and annular rupture. It has been shown that too low or high positioning can affect paravalvular regurgitation; too high positions can also result in coronary obstruction, whereas too low positions can cause conduction problems [9]. An implanted valve should also work properly with adequate leaflet coaptation and maximum effective orifice area for maintaining smooth hemodynamics with no transvalvular regurgitation, with normal levels of wall shear stress (WSS), and with normal levels of a transpulmonary pressure gradient. Maintaining normal WSS levels on the leaflets and aorta is vital for preventing degeneration of the tissue, as well as for preventing activation of platelets which might result in the generation of microemboli and thrombus. Maintaining normal pressure gradients is important for preventing fatigue failure of the valve and preventing failure risk of the left ventricle [10].

For a successful TAVR to prevent the complications listed above and to ensure the proper functioning of the valve, it is critically important to predict the performance of BHV upon implantation. As there is no direct view or access to the affected anatomy, accurate preoperative planning is crucial for a successful outcome. The most important decisions during planning are selecting the proper implant type/size and positioning of the valve. Due to the wide variety in device sizes and types and non-circular annulus shapes, there is often no obvious choice for a specific patient and most clinicians base their final decision on their previous experience. However, it is desirable to mechanically assess selected BHV before implantation to ensure proper valve function after implantation. Mechanical assessment refers to being able to realistically simulate the opening and closing of the valve to examine the valve function. Such an assessment will prevent improper valve selection that can lead to possible complications. While current practice for clinicians is performing an anatomical examination of the aorta geometry for optimal valve selection, more recently, CFD approaches are being evaluated to look at different scenarios of valve operation before implantation. Below, we explain the conventional medical image-based approach as well as the new CFD approach for TAVR.

## 3. Medical Imaging for TAVR

A wide range of imaging technologies and techniques is used to access heart conditions and obtain an accurate understanding of the cardiovascular pathology [11]. Cardiovascular imaging can be categorized into invasive and non-invasive techniques. Invasive imagining is a particularly relevant specialized modality during procedures and surgical interventions, such as inter-cardiac echocardiography for catheter-based procedures, as it facilities real-time tracking of catheter locations and early prediction of complications [12]. On the other hand, non- or minimally invasive cardiovascular imaging is commonly used for heart assessment and treatment planning of complex surgical interventions, including echocardiography, cardiac computed tomography (CT) scans, and cardiac magnetic resonance imaging (MRI).

Echocardiography (ECHO) provides information on the size, morphology, and functions of heart valves and chambers. Being cost-effective and safe, ECHO is often the starting point in managing cardiovascular diseases. Doppler may also be combined with ECHO to show heart regions of poor blood supply. However, the quantification of blood flow in ECHO relies on simplified assumptions based on local 2D flow vectors from low-resolution temporal and spatial image features. Thus, it is highly sensitive to noise, acquisition parameters’ values, and inter-operator variability [13,14]. Recently, 3D transesophageal (3D TEE) and transthoracic echocardiography (3D TTE) are becoming widely available due to advances in transducer technology. Three-dimensional TEE has proven beneficial in real-time surgical guidance for procedures such as mitral valve repair [15,16]. Moreover, 3D echocardiography can help in post-operative assessment where it can be used to calculate mechanical strain, providing a mechanistic understanding of left ventricle dysfunction before and after cardiac surgery [17].

In comparison with ECHO, cardiac CT facilitates the contrast differentiation between blood and tissues with superior spatial resolution. However, CT scans are relatively expensive, and this limits their use in resource-constrained settings [18]. Besides, patients get exposed to varying radiation levels depending on the CT protocol [19]. In terms of clinical applications, cardiac CT is a routine test for planning TAVR [20]. Moreover, CT is widely used for accurate 3D reconstruction of major heart valves and arteries [21,22,23]. Recently, cardiac computed tomography angiography (CCTA), together with cardiac MRI, have been utilized to generate high-fidelity virtual simulations for cardiovascular procedures, which can help optimize the approaches for surgery before hospitalization [24]. Multidetector CT along with transthoracic/transoesophageal echocardiography plays a key role in the pre-operative assessment of suitability for TAVR in measuring the size of aortic annuals, assessing the amount of calcification in the aortic root, and predicting the angle of deployment. CT accurately detects the aortic root size to select the proper valve size to avoid the complication of an undersized or oversized valve. Pre-operative CT also helps clinicians in the selection of route access. Any incidental findings in the chest, abdomen, or pelvis, such as a hidden malignancy, may affect the decision of TAVR. 

During the procedure, accurate positioning of the valve during TAVR is achieved using TEE echocardiography along with fluoroscopy. TEE is used immediately after deployment to assess the location and severity of aortic regurgitation. TEE is also used to detect post-operative complications, such as impairment of the coronary ostia, and also to detect any suspicion of coronary compromise.

MRI is a rapidly advancing non-invasive imaging technique that enables precise evaluation of heart function. A cardiac MRI sequence provides enhanced temporal resolution compared to CT scans but at the cost of a more extended scanning time [25]. A main limitation of MRI is that it cannot be used for patients having electro-metal implants, such as defibrillators, pacemakers, cochlear implants, and insulin pumps [25,26]. Modern pacemakers and defibrillators have an MRI mode that can be turned on during MRI scanning and turned off after MRI acquisition [27], thereby mitigating this limitation. MRI can also be used as an alternative CT in pre-TAVR planning, specifically, for patients who have contradictions to iodinated contrast medium due to severe allergic reaction or severely impaired renal function. MRI can reliably assess the severity of aortic stenosis when there is a discrepancy between clinical findings and ECHO due to poor autistic window, low flow, or inability to perform stress echocardiography [28]. MRI can provide both 3D volumetric visualization of a still heart and 4D blood flows of a moving heart. While capturing the time-varying cardiac anatomy, MRI can also acquire images of intravascular hemodynamics. Hemodynamics refers to the physical study of the blood flowing, together with solid structures, through heart chambers, arteries, and veins. Recently developed MRI machines enable full 4D mapping of intravascular flows, providing a crucial function to assess the hemodynamics of CVD patients. 

Although 4D flow MRI images provide a full overview of blood flow inside the cardiovascular system, there are a few limitations associated with spatiotemporal resolution, velocity encoding, and signal-to-noise ratio (SNR). The current resolution for 4D flow MRI images is limited, which leads to inaccurate calculation of some hemodynamic parameters, such as WSS. To address this need, several studies have recently investigated the use of CFD in combination with 4D flow MRI to compute hemodynamics. In a CFD simulation, blood flow hemodynamic parameters, such as pressure and velocity fields, are computed by solving the continuity equation and Navier–Stokes equations within the regions of interest (ROI). Nevertheless, CFD simulations depend on the accurate geometry of the ROI and personalized inlet and outlet boundary conditions. Therefore, 4D MRI can complement CFD solutions to a certain extent. With an advantage over MRI, CFD can accurately theoretically model blood flow with unlimited spatiotemporal resolution. 

## 4. Computational Modeling for TAVR Procedures

The opening and closing of the aortic valve is a very dynamic and complex event from a fluid mechanical perspective. Computational modeling is a powerful biomedical research approach for simulating blood flow hemodynamics and tissue behavior, whereas direct observations provide limited information. Relevant to TAVR, computational models potentially allow the virtual implantation of multiple device sizes at different implantation depths for a specific patient to provide useful insights that facilitate decision-making by physicians. To obtain good accuracy and satisfy the conditions in the model boundaries, such as blood pressure and velocity profile, the mechanical properties of the aortic root and bioprosthetic valve need to be properly defined. The relevant physical differential equations are then numerically solved to simulate the implanted valve. The simulation results represent the possible operation outcomes and the predicted performance of the implanted valve. To be valid, simulations should reveal mechanical stress on the tissue structures, such as contact pressure and WSS on the aortic wall, and principal stress on the aortic leaflets and prosthetic valve, and should show blood flow profiles for assessing heart function. 

Numerical approaches are used to solve the equations of continuum mechanics for leaflet deformation and hemodynamics of blood flow. Relevant computational models can be categorized into three main classes: FEA, CFD, and fluid–structure interaction (FSI) analysis [29]. FEA models include only the structural domain (i.e., aortic root, native and prosthetic valve leaflets), and these analyses allow the investigation of the structures to be performed by solving the continuum mechanical equations. For FEA models, the effect of surrounding blood is considered by applying a transvalvular uniformly distributed pressure load to the leaflets as a boundary condition. This simplified model type neglects blood flow and, therefore, does not reliably simulate the dynamics of the valve. CFD simulations provide information on the pressure and velocity fields within the fluid domain by solving the continuity and Navier–Stokes equations. This approach excludes structural fields and, hence, does not allow an assessment of structures, such as the movement of leaflets. For an accurate dynamic analysis that incorporates blood flow during the cardiac cycle coupled with the structural mechanics of the valve, FSI analysis is required as it considers both the structural and the fluid flow domains. In these simulations, the load applied to the leaflets is the result of coupling between two domains, as in the real case, and, hence, FSI models are essential to accurately simulate the full dynamic behavior of a BHV. Only such advanced models will enable a comprehensive assessment of implanted (or to be implanted) BHVs. Due to the high complexities in computational modeling of FSI, most relevant works have either adapted the FEA or CFD approaches. 

FEA has been employed in the crimping procedure for virtual pre-deployment and re-coiling during the virtual deployment process to assess mechanical stress on a BHV’s stent structure [10,30,31,32,33,34,35,36,37,38,39]. FEA studies have also focused on determining the stent contact areas on walls for assessment of anchoring [31,40]. FEA also has been used to determine the contact pressures on aortic roots by the stent, and the degree of apposition between the prosthesis stent and aortic root to assess the risk of conduction problems [31,32,38,39,41,42,43,44,45,46,47]. Moreover, FEA has been utilized to assess the risk of tissue degeneration by computing the structural stresses on leaflets for different valve designs and different implant depth positioning [31,33,37,38,39,45,46,48]. CFD studies, on the other hand, have revealed blood flow dynamics, enabling assessment of valve function and identification of paravalvular leakages [10,35,39,40,41,49,50] for different valve designs and different implant depth positioning. 

**Figure 2:**
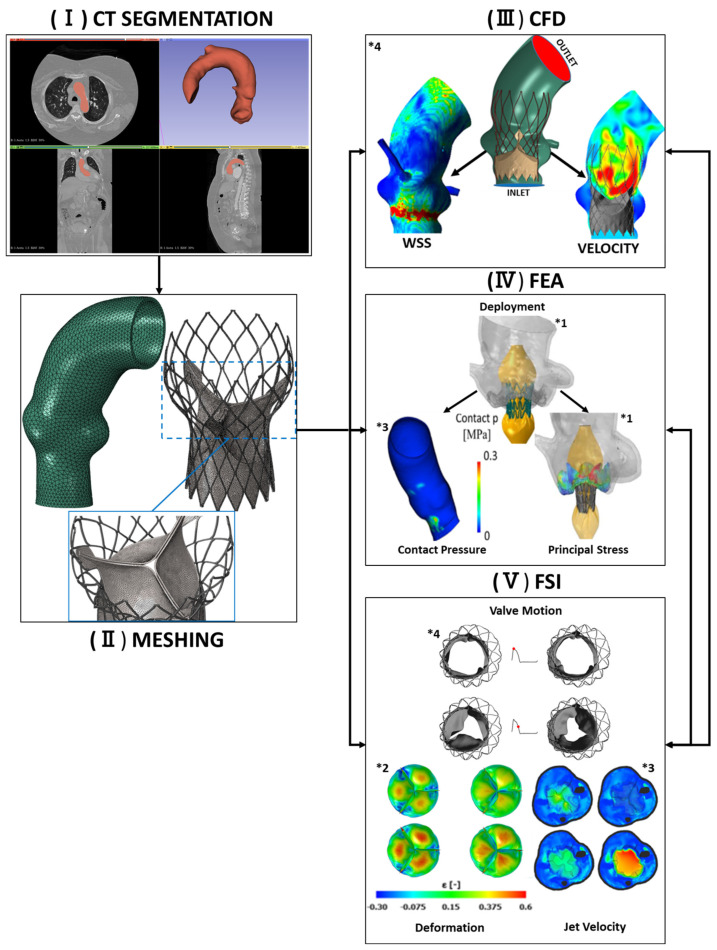
An end-to-end conventional computational modeling approach for assessing TAVR procedure: (**I**) patient-specific CT/MRI segmentation, (**II**) meshing, (**III**) CFD analysis, (**IV**) FEA modeling, and (**V**) FSI analysis. The figure captions are taken from *1 to *4 where they represent the following references [31,33,34,38], respectively.

While FEA and CFD studies provide limited information on structural stresses or flow hemodynamics, FSI analysis works on a comprehensive assessment of mechanical stresses on the BHV stent structure, aortic root, and the native and prosthetic leaflets caused by changing blood flow dynamics owing to valve motion [30,32,33,34,36,38,39,47,51,52,53]. An end-to-end conventional computational modeling approach for assessing the TAVR procedure is shown in Figure 2. Computational approaches are very powerful in assessing different valves for specific patients prior to TAVR, but their models suffer from long computational times and are not practical or readily available to clinicians. There is great potential that machine learning (ML) or DL approaches can expedite these models, which will be explained in the coming sections.

## 5. Deep Learning for TAVR Procedures

The immense development in DL techniques in recent years has led to state-of-the-art performances in various computer vision tasks, such as object detection, image classification, and image segmentation. This breakthrough has led to increased deployment of DL-based solutions across multiple life science fields, including the domain of biomedical health problems and complications. Specifically, convolutional neural network (CNN) has been proven extremely beneficial in numerous biomedical imaging applications, such as brain tumor detection [54], skin lesion classification [55], breast cancer detection [56], Alzheimer’s disease detection [57], and lung pathology screening [58]. 

**Figure 3:**
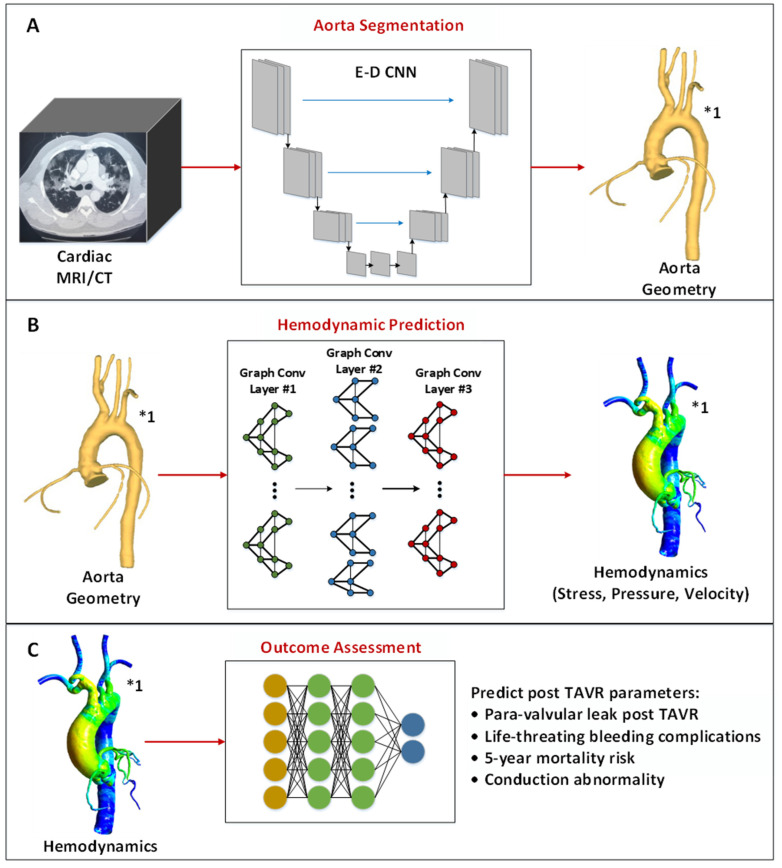
Graphical representation of related studies on DL alternatives for each stage of the conventional TAVR procedure planning approach including, (**A**) aorta segmentation, (**B**) hemodynamic prediction, and (**C**) pre- and post-operative clinical outcome assessments. The figure captions highlighted with “*1” are cited from the following study [77].

In recent years, DL has been widely investigated for various cardiac imaging applications, including diagnostic, prognostic, risk stratification, and therapeutic/surgical treatment planning [11]. Consequently, DL algorithms have been utilized across every aspect of cardiac image analysis, starting from efficient acquisition, segmentation, motion tracking, and disease classification to modeling genotype–phenotype interactions. Several studies have used deep architectures to enhance and reconstruct high-quality CT scans from lower-quality ones so that patients do not need to receive high radiation doses during the scanning process [59,60]. In a similar approach [61], encoder–decoder (E-D) CNN has been deployed for MRI super-resolution, enabling faster acquisition while maintaining low acquisition time. Besides, several studies have used DL algorithms for automatic segmentation of the heart [62], heart chambers [63], and major blood vessels (such as the aorta) [64]. Moreover, DL has been used for diagnosis, anomaly detection, and calcification scoring [65,66,67,68,69]. The authors in [65] proposed a three-stage DL system for early diagnosis of myocardial infarction from ECHO images. Velzen et al. [66,67] utilized a deep CNN model to automatically score coronary artery calcium in low-dose CT. Lessmann et al. [68] proposed a two-stage CNN architecture to detect coronary, thoracic aorta, and valvular calcification from low-dose CT. In [69], multi-task recurrent CNNs were deployed to detect and characterize coronary plaque types. In addition to diagnostic capabilities, machine learning (ML)/DL algorithms can assist clinicians in predicting mortality risk [70,71], intra-operative online prediction [72], longitudinal post-intervention monitoring of patients [73,74], and providing feedback and expediting rehabilitation [72,75,76]. On the contrary, just recently, DL algorithms have been investigated as a fast and computational light alternative to traditional CFD methods to compute patient-specific hemodynamics. In this section, we will provide a comprehensive review of current DL alternatives for each stage of the conventional approach, including aorta geometry segmentation from CT/MRI volumes, hemodynamic prediction, and pre- and post-operative clinical assessment. Figure 3 illustrates a graphical representation of related studies on DL alternatives for each stage of the conventional TAVR procedure planning approach, including (A) aorta segmentation, (B) hemodynamic prediction, and (C) pre- and post-operative clinical outcome assessments.

### 5.1. Aorta Segmentation Using Deep Learning 

In recent years, DL algorithms have been widely investigated for automatic segmentation of the heart [62], heart chambers [63], and major blood vessels such as the aorta [64]. Cardiac and aorta image-based segmented models derived from MRI/CT scans are being used clinically to simulate blood flow in the coronary arteries of individual patients to aid in the diagnosis of disease and in planning treatments and procedures, such as TAVR. Cheung et al. [78] utilized a compact variant of U-Net architecture to segment the aorta and coronary artery network from CTCA scans, achieving a dice similarity coefficient (DSC) of 91.2%. Shen et al. [79] optimized a 3D U-Net with an attention gate module to enhance the vessels’ segmentation while suppressing irrelevant regions, and their results achieved a 90.5% DSC. 

Moreover, numerous studies have investigated full heart segmentation by stratifying the seven heart substructures, including the ascending aorta (AA). The majority of these studies trained and evaluated DL models using the MM-WHS Challenge 2017 dataset [80], which comprises 20 labeled and 40 unlabeled CT volumes, as well as 20 labeled and 40 unlabeled MRI volumes. Liu et al. [81] utilized a two-stage 3D U-Net to first segment the whole heart by removing irrelevant lung and rib regions and then segment the heart’s main substructures. Superior aorta segmentation performance was achieved, with a 95.5% DSC. Ye et al. [82] incorporated multi-depth fusion with 3D U-Net to better extract context information, reaching a 96.7% DSC. Wang et al. [83] introduced different modified attention mechanisms to lead 3D U-Nets to focus on more salient information. The joint attention gate (AG) and U-CliqueNet (UCNet) modules showed the best performance for aorta segmentation, with a 96.8% DSC. 

On the other hand, creating ground-truth cardiac segmentation masks to train DL models is a tedious task, which also suffers from high inter- and intra-observer variability. Therefore, Vesal et al. [84] proposed a novel unsupervised domain adaptation method (UAD) based on adversarial learning to leverage source-domain labeled CT data to generate labels (masks) for unlabeled target-domain MRI data. Wang et al. [62] proposed a few-shot learning framework where semi-supervised approaches are utilized along with a self-training strategy for whole heart segmentation, with only 4 labeled CT scans being used for training and 16 CT scans being used for testing. The model showed a DSC value of 94.3% to segment the aorta region. A brief comparison of related studies on aorta segmentation is shown in Table 1.

### 5.2. Cardiovascular Hemodynamic Prediction Using Deep Learning

#### 5.2.1. Utilized Dataset 

Although CFD is a primary tool to model cardiovascular hemodynamics, the high computational costs and the complex operation of patient-specific computational analysis hinder its application. Recently, numerous studies started to deploy DL to predict the hemodynamics of various parts of the cardiovascular system [77,85,86,87,88,89,90,91], such as the aorta, coronary arteries, and left atrial appendage. DL models need a large and diverse amount of data to ensure good generalization performance on unseen data and to model the complex relationship between blood vessel shapes and hemodynamics. Most of these studies utilized limited clinical data from a cohort with only a few hundred CT/MRI records from patients/healthy subjects. Consequently, to increase the dataset size, synthetic cardiac geometries have been generated using statistical shape modeling (SSM) based on principal component analysis (PCA); the full approach is detailed in [92,93]. By varying the most meaningful eigenvalues with SSM models, synthetic geometries are ensured to capture significant shape variations, such as the overall size change, diameter variation, and curvature vibration. Liang et al. [85,86] generated 729 synthetic aorta geometries using SSM based on 25 CT scan records from ascending aortic aneurysm (AsAA) patients. The authors in [87,88] generated 300 left atrial appendage (LAA) virtual geometries using 103 real cardiac CT records. The authors in [89] created 3000 geometries based on 228 3D MRI records. Additionally, the flow boundary conditions from 87 4D MRI records were utilized with statistical distribution modeling (SDM) to obtain the inlet vector field for synthetic geometries. Li et al. [77] synthesized 1110 aorta geometries based on 110 CT scans for patients with LAD stenosis before and after coronary artery bypass surgery. A brief comparison of the utilized datasets in related studies is tabulated in Table 2. 

#### 5.2.2. Achieved Results

A detailed comparison between available studies for predicting hemodynamics using DL approaches is presented in Table 3. Liang et al. [85] proposed a DL model to directly estimate the stress distributions of the aorta for AsAA patients. They developed a fully connected neural network (FCN) with an autoencoder structure, which can be used as a surrogate to FEA where it can compute stress distributions a few orders of magnitude faster than FEA, with an average error of 0.891% and 0.492% in the peak von Mises stress and von Mises stress distributions, respectively. In a continuation of the previous work [85], Liang et al. [86] trained and evaluated an FCN to estimate the steady-state distributions of pressure and flow velocity inside the thoracic aorta as a fast alternative to CFD. The trained model was capable of predicting the pressure field with an average error of 1.427% and the velocity magnitude field with an average of 1.961%. Filipovic et al. [94] investigated different regressor models to predict maximal wall shear stress (MWSS) for AsAA patients, including multilayer perceptron (MLP), multilinear regression (MLR), partial least square (PLS), and recursive partitioning and regression trees (RPART), where MLP achieved the best results with a 0.001 RMSE value.

Morales et al. [87] investigated the hemodynamics of more complex geometry, LAA, where the prediction of endothelial cell activation (ECAP) could help assess the risk of thrombosis. Two DL models were proposed: a naïve neural FCN and a PCA-FCN model, where PCA helped to reduce the dimensionality and produce a more compact model. Both models predicted the ECAP reliably, with an average error of 4.72% and 5.75% for FCN and PCA-FCN, respectively. To further enhance the performance and accelerate the inference time, Pinilla [88] utilized CNNs to predict the hemodynamics of LLA geometry. An encoder–decoder (E-D) CNN was deployed, showing higher performance with an average error of 0.63% when compared to 0.73% for the PCA-FCN model [87] over a dataset of 206 synthetic LAA geometries. Moreover, the E-D CNN exhibited better classification performance with 87.9% accuracy when compared to 81.7% for the PCA-FCN [87]. Gharleghi et al. [95] proposed a deep CNN model to estimate the time-averaged wall shear stress (TAWSS) in coronary artery geometry, achieving an NMAE of 10.38%. Yevtushenko et al. [89] proposed a deep architecture that combines MLP, long short-term memory (LSTM), and 1D DenseNet to model the hemodynamics of patients with aortic coarctation using centerline aggregated (i.e., locally averaged) geometries. Farajtabar et al. [90] proposed an artificial neural network (ANN) to predict the velocity and pressure fields inside a coronary arterial network with the presence and absence of abnormalities, such as stenosis. The model showed reliable performance with average prediction accuracies of 98.7% and 93.2% for the pressure and velocity magnitudes, respectively. However, unlike previous studies where the hemodynamics of the entire geometry is predicted in a single forward pass, the proposed model in [90] predicts the hemodynamics for a single point in the arterial network one at a time; hence, it needs a larger inference time, thus making it less applicable for time-sensitive applications. Besides, hand-crafted features are used as the input to the network, unlike previous approaches where the model learns the optimum set of features from the input geometry throughout the training process. Jordanski et al. [97] investigated different alternatives for transient analysis of WSS distribution at different time points during the cardiac cycle for AsAA and carotid bifurcation geometries. Gaussian conditional random field (GCRF) achieved the best results, with a 0.93 and 0.95 coefficient of determination for the AsAA and carotid bifurcation models, respectively.

Li et al. [77] proposed a modified version of PointNet [101] to model the internal hemodynamics of aorta geometries for patients with LAD stenosis before and after coronary artery bypass surgery. The deployed architecture showed a high agreement with the CFD results, with an average prediction accuracy of 90% and a high computational efficiency by predicting the hemodynamics within one second, which was 600-fold faster than CFD for high-resolution aorta geometries with over 2 million nodes. The modified PointNet could effectively resolve the disorder of point clouds and introduce spatial relationships. 

Ferez et al. [91] compared a set of popular DL approaches to predict ECAP in LAA geometry, including FCN, E-D CNN, and graph convolutional network (GCN). GCN showed superior performance with a 0.521% mean absolute error (MAE) compared to 0.608%, 0.651%, and 0.654% MAE for PCA-FCN, ED-CNN with Cartesian mapping input, and ED-CNN with Bull’s eye mapping input, respectively. 

BHVs are commonly utilized for heart valve replacement, but they are prone to fatigue failure. Balu et al. [99] proposed an alternative DL-based finite element analysis to learn the deformation biomechanics of bioprosthetic aortic valves. An autoencoder-CNN was utilized to predict the final deformed, closed shape of the heart valve from the input aorta geometry with the original undeformed heart valve. The input boundary condition, together with the valve material property, was fused with the geometry embedded in the bottleneck layers. Additionally, the coaptation area, a key parameter to determine BHV health, was predicted directly using a single MLP (neuron) connected to the bottleneck path. With further development, the proposed tool can provide fast decision support for designing surgical bioprosthetic aortic valves. Oldenburg et al. [100] used a U-Net architecture to predict simplified 2D flow during peak systolic steady-state blood flow through mechanical aortic valves with varying opening angles in randomly generated aortic root geometries, achieving MSE values less than 0.06. 

#### 5.2.3. Deployed Models

The deployed DL architectures are described below, including MLP, PCA+ MLP, E-D CNN, and GCN. 

ANN or FCN is the key algorithm at the heart of DL algorithms, which uses an aggregate of MLP to understand and map the input data of one form into the desired output form. Different variants of FCN have been considered for hemodynamic prediction from cardiac geometries, including vanilla architecture with multiple hidden layers and autoencoder (AE)-NN. In the AE-NN architecture, the geometry data are first compressed into an encoded representation passing through several dense layers, followed by a max-pooling operation. Next, the shape code is mapped to the stress/pressure/velocity code using a few dense layers. Finally, the decoder network constructs the full stress distribution using the latent stress representation through consecutive dense layers and upsampling layers. 

Additionally, full geometry and PCA-reduced geometry data have both been considered for training DL models. PCA remains a key algorithm for dimensionality reduction of data, thereby increasing interpretability while minimizing information loss. Nevertheless, PCA requires all geometries to be registered to a common template. Therefore, in [91], a non-rigid volumetric registration was applied to 3D cardiac geometries, following the approach in [102], to register all meshes to a fixed number of nodes. Afterward, non-linear mapping between the low dimensional representation of cardiac morphology and the corresponding CFD results was computed through FCN models. The PCA-FCN approaches showed slightly lower results compared to the conventional neural network (NN) while providing a much shallower architecture, which significantly reduced the computational burden.

Moreover, CNN has also been investigated to predict cardiac hemodynamics [88]. Convolutional filters are the main building blocks of CNNs, having several advantages when compared to conventional NN, such as limited connections and weight sharing, which provides increased learning capabilities, enabling the network to automatically extract useful features without any human supervision. However, CNNs deal with 1D, 2D, or 3D Euclidian structured data, such as acoustic signals, images, or videos. Therefore, to train CNN models on non-Euclidean structured data (3D geometries), the data must be first transformed to Euclidean representation (2D planes) [88].

Furthermore, a state-of-the-art DL algorithm, graph convolutional network (GCN), has been compared with the aforementioned DL approaches to predict ECAP in LAA geometry [91]. The recently emerged GCN operates over non-Euclidean irregular geometries with a varying number of nodes, while FCN and E-D CNN require previous registration or 2D mapping of the input to a common template with a fixed number of mesh nodes. Consequently, GCN predicts the overall ECAP distribution based solely on anatomical features and shows superior performance compared to the counterpart DL approaches. 

### 5.3. Outcome Assessment of TAVR Procedures Using Machine/Deep Learning

Several studies have investigated DL algorithms for assessing the outcomes of TAVR procedures, predicting post-operative complications such as late major bleeding, and predicting long-term mortality risk. Table 4 compares these studies in terms of the utilized datasets, targeted problems, deployed DL models, and achieved results. Wang et al. [103] proposed a compact 3-layer CNN classification model to predict paravalvular leak (PVL) post TAVR, which is a major complication of the TAVR procedure. The proposed model predicted PVL from post-operative CT data, achieving sensitivity and specificity values of 76.91% and 86.88%, respectively. Jia et al. [104] introduced BLeNet, a risk prediction model, to predict major or life-threatening bleeding complications (MLBCs) after TAVAR. BLeNet outperformed two standard survival analysis models, the traditional Cox proportional hazard (Cox-PH) and the random survival forest models, achieving a value of 0.84 for the area under the curve (AUC) compared to 0.72 and 0.70 AUC for the Cox-PH and survival forest models, respectively. BLeNet was developed with 56 baseline procedural and post-procedural characteristics, including lab tests, CT characteristics, echocardiographic features, procedural details, and antithrombotic medications. Moreover, Pesno et al. [105] used a DL approach to predict the 5-year mortality risk after TAVR based on several clinical and echocardiographic variables, achieving 0.79 AUC and 71% sensitivity values. Agasthi et al. [106] utilized a gradient boosting (GB) classifier to predict 1-year mortality after TAVR. Baseline demographics, ECG, CT scans, and ECHO data from 1055 patients were used to train the ML model, where it achieved an AUC of 0.72, outperforming two traditional surgical risk scores, TAVR-SCORE and CoreValve, which had AUC of 0.56 and 0.53, respectively. 

On the other hand, Galli et al. [107] proposed combined mechanistic modeling and an ML approach for patient-specific prediction of conduction abnormalities after TAVR. Virtual valve implementation for several valve designs was performed for 151 patients using FEA, and then the derived mechanistic variables along with the anatomical variables and procedural variables (prosthetic valve type and size) were used to train multiple ML models. The homogenous ensemble of all models using bootstrap aggregation showed the best performance for predicting post-operative conduction abnormalities with AUC, sensitivity, and specificity of 0.84, 100%, and 62%, respectively. This shows the potential of the synergetic approach for personalizing procedure planning, allowing the selection of optimal prosthetic valve and implantation strategy and avoiding new conduction abnormalities. Moreover, Astudillo et al. [108] proposed an automated DL-based method to predict aortic annulus perimeter and area from plane aortic annular CT images. A U-Net model was used to predict aortic annulus regions, followed by a post-processing step to compute the area and perimeter. The proposed method showed similar results compared to two independent medical observers, proving that it could be incorporated into pre-operative TAVR planning routine as a fast and accurate device size selection method.

## 6. Future Directions 

After analyzing critically and concisely earlier research in the literature, it can be seen that DL approaches have recently been utilized in the cardiovascular biomechanics field; more specifically, a few works only targeted pre-operative planning and post-operative assessment of the TAVR procedure. Consequently, there is room for improvement to overcome the current gaps and limitations. Individual works have targeted specific tasks, such as aorta geometry segmentation from cardiac imaging, hemodynamic prediction, or pre- and post-operative clinical assessments. However, no work has proposed an end-to-end DL pipeline that integrates all three stages in a single framework. Besides, the work performed for each stage has several limitations, which can be further improved, as described in the upcoming paragraphs. 

Medical image segmentation is a well-matured domain where rapid advancements during the last few years with DL segmentation models, specifically the variants of ED-CNNs such as U-Net models, have yielded state-of-the-art results (>95%) for cardiac and aorta segmentation problems. On the other hand, DL approaches have just recently been proposed as a surrogate for hemodynamic modeling. Among several DL alternatives, it has been proven that GCNs exhibit the best performance, empowered by their capability to operate directly over non-Euclidean irregular geometries with a variable number of mesh nodes. Thus, GCNs can learn solely based on anatomical features and present the best performance among various DL approaches. With GCNs being a new DL domain, there is room for investigating different variants of the model with the aim of proposing modified architectures tailored for the computational modeling process. Furthermore, numerous studies have investigated DL and classical ML approaches for assessing TAVR outcomes, predicting post-operative complications, and predicting prosthetic valve defects. However, GCNs are yet to be investigated for the sole outcome prediction stage. 

Nonetheless, more compact architectures can be developed at each stage of the TAVR DL pipeline to speed up the inference process while minimizing the computational complexity. This can be achieved using the new generation of heterogeneous models based on self-organized operational neural networks (Self-ONNs) [109,110]. As a future direction, non-linear operational neurons can be incorporated with GCN architectures to enhance learning capabilities and further boost performance. 

On the other hand, to enhance the generalization performance of DL models, there is a need for larger and more diverse cohort aorta CT/MRI datasets that encapsulate various aorta shapes and sizes from different age groups, genders, and ethnicities around the world. As mentioned in the previous sections, current studies have used up to a few hundred cases only, where it goes up to a few thousand by augmentation. The majority of current studies have utilized the SSM approach for data augmentation to generate synthetic aorta geometries. However, advanced approaches such as deep generative models (i.e., generative adversarial networks and denoising diffusion probabilistic models) can be utilized for the data augmentation tasks, which can significantly enhance the performance [111,112,113,114]. Moreover, high-resolution meshed aorta geometries with a few million nodes are needed to generate reliable computational modeling results. Surface nodes, which can go up to a few hundred thousand nodes, can be used to train DL models to generate CFD/FEA results. However, the entire solid geometry with millions of nodes must be used to train a DL FSI surrogate model. Therefore, none of the previous studies have investigated DL for FSI analysis due to the significant computational burden. The patch-based approach is a potential solution where the input geometry is divided into smaller geometry patches before being fed to a DL model, while the final FSI result is created by merging the individual patch results. Furthermore, the concept of super-resolution [115] can be deployed using a two-stage DL approach, where, first, a DL model generates FSI results from lower-resolution input geometries, and then a second DL model increases the resolution of the generated FSI result. To overcome the huge computation burdens of processing large mesh data, Strönisch et al. [116] proposed a multi-GPU approach for training GCNs. This approach scales the state-of-the-art computational modeling surrogate DL models from the domain of graph-based machine learning to industry-relevant mesh sizes for numerical flow simulation.

The majority, if not all previous TAVR DL approaches, have focused on steady-state analyses of CFD parameters, such as velocity, wall shear stress, and pressure, or FEA parameters, such as mechanical stress on the aortic native and prosthetic aortic valve, aortic root, and BAH stent, whereas transient assessment of these parameters has barely been obtained with DL methods. Steady analysis is non-practicable in time-dependent and complex FSI analyses, such as aortic valve analysis with moving leaflets, owing to the cardiac cycle and the moving nature of the prosthetic valve. Besides that, more complex analyses, such as virtual BAH implantation and valve movements associated with the FSI analysis of the implanted BAH, have never been investigated using DL approaches. Moreover, derived parameters obtained by these complex analyses, such as paravalvular leakage, flow velocity, and mechanical stress on the native and prosthetic aortic valves, aortic root, and BAH stent, have barely been investigated using DL techniques. 

**Figure 4:**
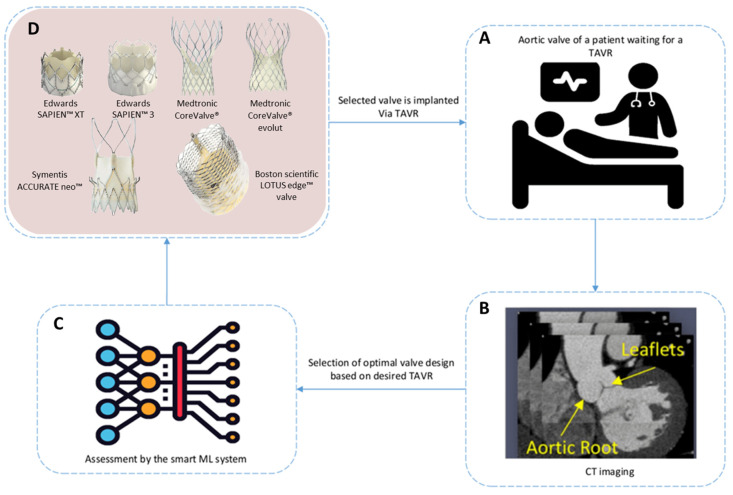
The proposed end-to-end smart DL framework for real-time assessment and recommendation of the best prosthetic valve (BHV) design for the TAVR procedure. (**A**) Admitted patient waiting for TAVR surgery, (**B**) CT scans for aortic root, showing the location of the valve to be implanted, and (**C**,**D**) virtual implementation and assessment of different valve designs [117] using the DL-based system to recommend the best valve for the surgery.

As a future direction, an end-to-end smart DL framework is outlined for real-time assessment and recommendation of the best prosthetic valve design for the TAVR procedure, as shown in Figure 4. This framework summarizes and builds on the individual efforts of earlier studies. It provides a unified solution that can be implemented and further enhanced in future studies. First, during the training phase, FEA can be performed for the virtual deployment of different valve designs using segmented patient-specific cardiac imaging data (i.e., CT/MRI) and BHV geometries. Next, FSI analysis can be performed by combining CFD and FAE analyses. Afterward, numerous critical mechanical and hemodynamic parameters that help in selecting the optimal BHV design can be derived from the transient FSI analysis. Finally, a three-stage DL framework can be trained using the generated data where, first, a compact ED-CNN segments the aorta and BHV geometries from the input CT scan. Next, three different regression GCNs can be utilized to generate CFD, FEA, and FSI results. Finally, a compact classification GCN can be trained to automatically generate critical mechanical/hemodynamic parameters and to recommend the best valve design. Moreover, an additional model can be deployed to predict any potential complications of the TAVR procedure for each selected valve design. The final DL system can automatically assess different BHV designs to identify the optimal BHV for specific clinical TAVR cases, thereby supporting clinicians in minimizing risks during TAVR therapy planning.

## 7. Conclusions

BHVs are commonly used as heart valve replacement via the TAVR procedure; however, BHVs are prone to fatigue, and estimating their remaining life directly from medical imaging is difficult. Besides, it is of utmost importance to select the optimal valve design among several alternatives for each specific TAVR case. In recent years, CFD, FEA, and FSI analysis have emerged as effective tools to analyze valve performance, providing better guidance for personalized valve design. Nevertheless, such patient-specific computational modeling requires long computational time and complex procedures. On the other hand, DL can offer a computationally light surrogate that can render the hemodynamic parameters in a few seconds, providing a real-time clinical solution. In this work, we provided a comprehensive review of medical imaging, conventional computational modeling, and DL approaches for TAVR planning and outcome assessment. We mainly focused on DL approaches, comparing related studies in terms of the utilized datasets, augmentation techniques, deployed DL models, and achieved results. Based on the conducted review, among different DL models, GCN can be the most appropriate alternative for assessing TAVR procedures’ outcomes, given it is ability to operate directly on irregular non-uniform aorta geometries. Previous studies have utilized GCN to regress hemodynamics in various parts of the cardiovascular system, such as the aorta. However, GCN deployment can be extended in future studies to classification tasks to recommend the best valve design or to predict any possible post-operative complications. Moreover, data scarcity is a key issue for DL-based TAVR assessment systems. Therefore, creating larger and more diverse datasets with pairs of CT/MRI images and their corresponding computational modeling simulation results can ease the training process and further enhance the generalization performance of DL models. Moreover, advanced deep generative models, such as generative adversarial networks and denoising diffusion probabilistic models, can be utilized for further data augmentation. Furthermore, we outlined an end-to-end smart DL framework that can be implemented in future studies for real-time assessment and recommendation of the best BHV design for TAVR. 

## Figures and Tables

**Table 1:** Comparison between studies on aorta segmentation using deep learning methods in terms of utilized datasets, deployed models, and achieved results.

Ref.	Dataset		DL Model	Results
	3D CT/MRI Scans	2D Slices		
[78]	69 CT Scans	14,597	2D U-Net	DSC 91.2%
[79]	70 CT Scans	11,200	Attention Gate 3D U-Net	DSC 90.5%
[81]	20 CT and 20 MRI (MM-WHS Challenge 2017)	_	Two-Stage 3D U-Net	DSC 95.5%
[82]	MM-WHS Challenge 2017	_	Multi-Depth Fusion U-Net	DSC 96.7%
[83]	MM-WHS Challenge 2017	_	AG-UCNet	DSC 96.8%
[84]	MM-WHS Challenge 2017	_	UAD	DSC 81.3%
[62]	MM-WHS Challenge 2017	_	Few-Shot Learning Framework	DSC 94.3%

**Table 2:** Comparison between studies for predicting hemodynamics using deep learning approaches in terms of utilized datasets, synthetic datasets, and whether the dataset is available publicly.

Ref.	Dataset	Synthetic Dataset	Availability
[85]	25 CT scans from AsAA patients	729 geometries created using SSM based on PCA [92,93]	√
[86]	Similar to [85]	Similar to [85]	√
[94]	NA	6000 geometries based on different parameters (i.e., AsAA length and curvature)	
[87]	103 cardiac CT scans	300 LAA geometries created using SSM based on PCA	√
[88]	Similar to [87]	Similar to [87]	√
[95]	127 coronary artery CT scans	3302 modified bifurcation geometries based on generic shape change [96]	
[89]	- 143 3D MRI scans from CoA Patients - 85 healthy 3D MRI scans - 87 4D MRI scans to obtain flow boundary conditions	3000 aortic geometries and inlet vector fields created using SDM	
[90]	120 coronary arterial geometries (with/without stenosis)		
[97]	NA	4000 AsAA and carotid bifurcation 2D geometries created using in-house software [98]	
[77]	110 CT scans from patients with LAD stenosis	1110 geometries	
[91]	- 256 synthetic and real LAA geometries - 114 real LAA geometries		
[99]	90,941 valve closure simulations		
[100]	3500 mechanical aortic valves with varying opening angles in randomly generated aortic root geometries		

**Table 3:** Comparison between studies for predicting hemodynamics using deep learning approaches in terms of computational modeling used to generate the target results to train DL models, the input and output of the DL models, the deployed DL models, and the achieved results.

Ref	Computational Modeling	Input Geometry	Output	DL Model	Predicted Hemodynamic Results	Derived Parameter Results
[85]	FEA	Aorta geometries	Aortic wall stress distributions	PCA + MLP	Estimated stress distribution: NMAE_S11_ of 0.492%, NMAE_S22_ of 0.492%, and NMAE_S12_ of 0.492% - Estimated peak stress value: NMAE_S11_ of 0.891%, NMAE_S22_ of 0.891%, and NMAE_S12_ of 0.891%	Estimated stress distribution: NMAE_Von Mises_ of 0.492% - Estimated peak stress value: NAE_Von Mises_ of 0.891%
[86]	CFD	Aorta geometries	Pressure field and velocity field magnitudes	MLP	- Pressure field: NMAE of 1.427% - Velocity magnitude: NMAE of 1.961%	
[94]	CFD	Aorta geometries	MWSS	(1) MLR (2) PLS (3) RPART (4) MLP	- MLP: MAE of 0.001%	
[87]	CFD	LAA geometry	ECAP map	(1) MLP (2) PCA + MLP	- MLP: MAE of 0.646% and NMAE of 4.720% - PCA + MLP: MAE of 0.649% and NMAE of 5.756%	Binary classification based on ECAP values (subject at risk or safe)
[88]	CFD	LAA geometry	ECAP map	(1) PCA + MLP (2) ED-CNN	- PCA + MLP: MAE of 0.73% - E-D CNN: MAE of 0.63%	Binary classification based on ECAP values: - PCA + MLP MAE 81.7% - E-D CNN MAE 87.9%
[95]	CFD	Coronary artery	TAWSS	CNN	NMAE of 10.38%	
[89]	CFD	Center-line based shape model and flow boundary conditions	WSS, SFD, and KE along the centerline	Encoder NN +LSTM +1D DenseNet		
[90]	CFD	Hand-crafted features for each node in the coronary artery geometry	Pressure field and velocity field magnitude at a specific node	MLP	- Pressure field accuracy of 98.7% - Velocity magnitude accuracy of 93.2%	
[97]	CFD	2D geometry of AsAA and carotid bifurcation	WSS	GCRF	- AsAA coefficient of determination of 0.93 - Carotid bifurcation coefficient of determination of 0.95	
[77]	CFD	Geometry containing aorta, coronary arteries, and bypass graft	3D pre-operative and post-operative velocity and pressure field	PointNet	Aorta and superior aortic branch artery - Pre-operative values: NMAE_Pressure_ of 4.30% and NMAE_Velocity_ of 6.01% - Post-operative values: NMAE_Pressure_ of 4.28% and NMAE_Velocity_ of 6.02%	
[91]	CFD	LAA geometry	ECAP	(1) PCA + MLP (2) E-D CNN (3) Geometric PointNet	- PCA-MLP MAE of 0.608% - ED-CNN with Cartesian mapping input: MAE of 0.651% - ED-CNN with Bull’s eye mapping input: MAE of 0.654% - Geometric CNN: MAE of 0.521%	
[99]	FEA	- Aorta geometry with undeformed heart valve - Aortic pressure - Valve material property	- Deformed, closed shape of the heart valve - Coaptation area	Autoencoder-CNN	- Valve deformation: Euclidean distance of 0.0649 cm - Coaptation area: CC of 0.933 - RMSE of 0.117 cm^2^	
[100]	CFD	Aortic root with mechanical valve	Pressure field and velocity field magnitude	U-Net	MSE < 0.06	

**Table 4:** Comparison between studies for outcome assessment of TAVR procedures using deep learning methods in terms of the utilized datasets, targeted problems, deployed models, and achieved results.

Ref.	Dataset	Targeted Problem	DL Model	Results
[103]	168 CT scans	PVL prediction	3-layer CNN	Sens. 76.9% Spec. 86.9% Acc. 78.6%
[104]	668 imaging scans and clinical data	MLBC prediction	2-layer FCN (BLeNet)	AU 0.84 3-Year Sens. 67% 3-Year Spec. 89%
[105]	471 ECHO scans and clinical data	5-year mortality prediction	FCN	AU 0.79 Sens. 71%
[106]	Demographics, ECG, CT scans, and ECHO data from 1055 patients	1-year mortality prediction	Gradient boosting	AUC 0.72
[107]	151 CT scans	Conduction abnormality prediction and best valve design recommendation	Ensemble of ML models	AU 0.84 Sens. 100% Spec. 62%
[108]	453 CT scans	Aortic annulus perimeter and area	U-Net	Area MSE 0.1089 cm^2^ Perimeter MSE 0.6 cm

## Data Availability

No new data were created or analyzed in this study. Data sharing is not applicable to this article.

## Abbreviations

TAVR	Transcatheter Aortic Valve Replacement
TAVI	Transcatheter Aortic Valve Implantation
AV	Aortic Valve
BHV	Bioprosthetic Heart Valve
CFD	Computational Fluid Dynamics
FEA	Finite Element Analysis
FSI	Fluid–Solid Interaction
DL	Deep Learning
ML	Machine Learning
GCN	Graph Convolutional Network
CNN	Convolutional Neural Network
E-D CNN	Encoder–Decoder CNN
CVD	Cardiovascular Disease
WHO	World Health Organization
MRI	Magnetic Resonance Imaging
CT	Computed Tomography
CCTA	Cardiac Computed Tomography Angiography
ECHO	Echocardiography
3D TEE	3D Transesophageal Echocardiography
3D TTE	Transthoracic Echocardiography
PARTNER	Placement of Aortic Transcatheter Valve
AA	Ascending Aorta
AsAA	Ascending Aortic Aneurysm
CoA	Coarctation of the Aorta
LAD artery	Left Anterior Descending artery
LAA	Left Atrial Appendage
SSM	Statistical Shape Modeling
SDM	Statistical Distribution Modeling
PCA	Principal Component Analysis
MWSS,	Maximal Wall Shear Stress
TAWSS	Time-Averaged Wall Shaer Stress
WSS	Wall Shear Stress
SFD	Secondary Flow Degree
KE	Kinetic Energy
ECAP	Endothelial Cell Activation Pressure
AG-UCNet	Attention-Gate U-CliqueNet
UAD	Unsupervised Domain Adaptation
MLP	Multilayer Perceptron
MLR	Multilinear Regression
FCN	Fully Connected Neural Network
PLS	Partial Least Square
RPART	Recursive Partitioning and Regression Trees
LSTM	Long Short-Term Memory
Self-ONN	Self-Organized Operational Neural Network
GAN	Generative Adversarial Networks
GPU	Graphics Processing Units
DSC	Dice Similarity Coefficient
MAE	Mean Absolute Error
NMAE	Normalized Mean Absolute Error
RMSE	Root Mean Squared Error
MSE	Mean Squared Error
PVL	Paravalvular Leak
MLBCs	Major or Life-Threatening Bleeding Complications
